# On the Use of a Test to Exhaustion Specific to Tennis (TEST) with Ball Hitting by Elite Players

**DOI:** 10.1371/journal.pone.0152389

**Published:** 2016-04-01

**Authors:** Cyril Brechbuhl, Olivier Girard, Grégoire P. Millet, Laurent Schmitt

**Affiliations:** 1 National Technical Direction, French Tennis Federation, Paris, France; 2 Department of Physiology, Institute of Sport Sciences (ISSUL), Faculty of Biology and Medicine, University of Lausanne, Lausanne, Switzerland; 3 Department of Research and Performance, National Centre of Nordic-Ski, Premanon, France; University of Rome Foro Italico, ITALY

## Abstract

**Purpose:**

We aimed to a) introduce a new Test to Exhaustion Specific to Tennis (TEST) and compare performance (test duration) and physiological responses to those obtained during the 20-m multistage shuttle test (MSST), and b) determine to which extent those variables correlate with performance level (tennis competitive ranking) for both test procedures.

**Methods:**

Twenty-seven junior players (8 males, 19 females) members of the national teams of the French Tennis Federation completed MSST and TEST, including elements of the game (ball hitting, intermittent activity, lateral displacement), in a randomized order. Cardiorespiratory responses were compared at submaximal (respiratory compensation point) and maximal loads between the two tests.

**Results:**

At the respiratory compensation point oxygen uptake (50.1 ± 4.7 *vs*. 47.5 ± 4.3 mL.min^-1^.kg^-1^, p = 0.02), but not minute ventilation and heart rate, was higher for TEST compared to MSST. However, load increment and physiological responses at exhaustion did not differ between the two tests. Players’ ranking correlated negatively with oxygen uptake measured at submaximal and maximal loads for both TEST (r = -0.41; p = 0.01 and -0.55; p = 0.004) and MSST (r = -0.38; P = 0.05 and -0.51; p = 0.1).

**Conclusion:**

Using TEST provides a tennis-specific assessment of aerobic fitness and may be used to prescribe aerobic exercise in a context more appropriate to the game than MSST. Results also indicate that VO_2_ values both at submaximal and maximal load reached during TEST and MSST are moderate predictors of players competitive ranking.

## Introduction

Tennis is a sport that requires a mixture of speed, agility, strength, and power combined with moderate-to-high aerobic and anaerobic capacities [1,2]. Although the sport-specific technical skills and tactical choices are predominant factors, players require a well-developed physical conditioning to execute advanced shots and maintain stroke efficiency as fatigue develops [3]. Evaluation of aerobic fitness is commonly used to characterize training effects, evaluate physical fitness, and identify target training areas [4]. When assessing players’ aerobic fitness level the ‘Gold-standard’ protocol is the direct measurement of maximal oxygen uptake ($\dot{\text{V}}$O_2max_) while running to exhaustion (i.e., progressive running speed increments) on a treadmill in a laboratory environment [5].

In tennis, a large variety of field running protocols, from which either indirect (i.e., estimation from performance achieved) or direct (i.e., using portable gas analyzer) $\dot{\text{V}}$O_2_ measurements are derived, have been popularized over the last 30 years [6,7]. The 20-m multistage shuttle test (MSST) is probably the most popular field procedure in the tennis community, and represents an integral part of the regular test battery of leading national tennis federations (US tennis association, Tennis Australia, French Tennis Federation) [1]. However, MSST is known to under-predict $\dot{\text{V}}$O_2max_ among racket-sport players [8]. Furthermore, it is only semi-specific to tennis, as it does not accurately reflect movement patterns that are typically performed on court. Hence, MSST cannot simulate the specific muscular involvement of the upper limbs with respect to ball hitting. Playing tennis also requires rapid directional changes after covering a distance of less than 5 m, yet players would only have to change direction every 20 m during MSST.

Over the past decade, field-based test protocols have been developed to evaluate aerobic fitness of tennis players in a context more appropriate to the game [1,9–12]. For instance, the Girard Test [11] and the Hit & Turn Tennis Test [10], two incremental protocols (i.e., stages duration of 40–50 s, interspersed by 10–20 s of rest and including movements’ speed/direction changes controlled by visual and/or auditory feedbacks) to exhaustion, have been popularized. The main drawback of these test procedures, however, is that background strokes are simulated (i.e., stroke mimic actions) so that there is actually no “hitting of the ball” (i.e., racket / ball contact). To our knowledge, there is no direct comparison in the literature of cardiorespiratory responses or energy expenditure during tennis tests performed with vs. without ball hitting. Moreover, large between-player differences regarding the intensity of the mimed strokes may thereby complicate standardization of these tests. This is not a trivial issue since the work of upper limbs, known to influence the overall energetic demand in tennis [13] would likely modify the physiological responses when players are required or not to hit the ball. For example, hitting backstrokes was shown to induce 2–10% higher physiological measurements than forehands [13,14], which increased with stroke velocities.

More recently, the Baiget Test [9] that includes ball hitting at increasing intensity from a throwing machine has been proposed. This test actually builds on the test originally developed by Smekal et al. [12]. In the original version of the “on-court” test [12] players began hitting balls at a ball frequency (BF) of 12 shots.min^-1^, which was further increased by 2 shots.min^-1^ every 3 minutes. Compared to this original test procedure [12], the main modifications incorporated in the Baiget Test were (a) the inclusion of shorter stages (3 vs. 2 minutes) and (b) a slower starting BF (12 vs. 9 shots.min^-1^) [9].These changes were likely implemented to limit test duration, which favors more accurate determination of the main cardiorespiratory parameters at specific time points (i.e., respiratory compensation point (RCP) and maximal load) when evaluating aerobic fitness. In the Baiget Test [9], however, the activity is continuous in nature without inclusion of any recovery breaks characterizing intermittent efforts in tennis. By comparing physiological responses between their field-based procedure and a discontinuous treadmill test, Girard et al. [11] highlighted that laboratory tests underestimate $\dot{\text{V}}$O_2max_ values, while cardiorespiratory variables measured at submaximal intensities did not differ. No such comparison has been made for the other available tennis-specific incremental procedures [9,10,12]. When it comes to estimate aerobic fitness of tennis players, determining whether physiological responses to a newly designed protocol are similar or not compared to a ‘reference’ test (MSST) is practically relevant.

In racket sports, as in every sport, the physiological responses are known differ between players of various playing standards. It is therefore informative for the coach to establish the ability of a test procedure to discriminate players of various standards. Although time to exhaustion between a squash and a treadmill test were not different only performance during the squash test correlated with players’ ranking [15]. More specifically, Baiget et al. [9] highlighted the usefulness of using technical effectiveness combined with physiological parameters at submaximal intensity (e.g respiratory compensation point, RCP) to best predict tennis performance level. Reporting the nature of the relationship of test duration and/or physiological outcomes with players’ competitive ranking is therefore crucial, for instance, to assess seasonal changes in players’ fitness.

Therefore, we aimed to a) introduce and validate a Test to Exhaustion Specific to Tennis (the so-called TEST) including elements of the game (i.e., actual ball hitting, lateral displacements, intermittent activity), b) compare TEST performance (test duration) and physiological responses (i.e., at RCP and maximal load) to those obtained during a ‘classical’ field procedure (MSST) highly popular in a large number of clubs and federations, and c) determine to which extent test duration and $\dot{\text{V}}$O_2_ values for both test procedures correlate with performance level (competitive ranking). We hypothesized that cardiorespiratory responses derived from TEST would be higher than MSST and that TEST would be a better predictor of players’ ranking.

## Methods

### Ethic Statement

Both the players and their parents (for minors) provided written informed consent for the study after the procedures and potential risks associated with participation in the study were fully explained. The scientific committee of the French Tennis Federation approved the study that was performed in accordance with the ethical standards reported [16], and conformed to the recommendations of the Declaration of Helsinki.

### Subjects

Twenty-seven high-level competitive tennis players (8 males and 19 females) (mean ± SD; age: 16.8 ± 0.9 years; height: 185.4 ± 5.3 cm; body mass: 75.8 ± 7.2 kg for males and age: 17.2 ± 2.4 years; height: 173.3 ± 9.9 cm; body mass: 64.7 ± 9.5 kg for females), volunteered to participate in the study. They were all members of the national teams of the French Tennis Federation (international tennis number (ITN): 1 (elite)). The mean training background of the players was 11.0 ± 3.5 years and the training regimen was 6 d.week^-1^ with a training volume of 24.0 ± 2.1 h.week^-1^. Players were focusing 15.1 ± 1.2 h.week^-1^ on tennis-specific training (i.e. technical and tactical skills), while their fitness routine also included aerobic and anaerobic (i.e. on-court and off- court exercises; 4.4 ± 0.3 h.week^-1^) conditioning as well as strength (5.2 ± 0.5 h.week^-1^) training. During the three months before testing (November), they participated regularly to official tennis competitions (i.e. “International Tennis Federation Juniors”, and “International Tennis Federation Futures” or ATP tournaments) for a total of 5–10 matches monthly.

### Experimental Design

All participants performed two incremental protocols to exhaustion in randomized order: the ‘traditional’ MSST [17] and a new Test to Exhaustion Specific to Tennis (the so-called TEST). Each test was conducted under similar standard environmental conditions (temperature ~ 20°C, relative humidity ~ 50%) at the same time of day (± 2h), 2 days apart, on an indoor tennis court (i.e., GreenSet surface, GreenSet Worldwide S.L., Barcelona, Spain). All participants were given written and verbal instructions to report for testing in a well-rested, well-hydrated and well-nourished state, and to refrain from eating at least two hours before testing. They were told to refrain from strenuous training the day before the test. All players were familiar with MSST as part of their regular (twice yearly) physical performance assessment. One week before the main experimental visits, all participants attended one familiarization session where TEST requirements were explained. At this occasion, players performed TEST (yet without mouth mask) so as to limit any learning effect.

### Experimental procedures

#### The 20-m multiple-shuttle-run test (MSST)

MSST procedure has been described elsewhere [17]. Briefly, participants ran back and forth on a 20-m course, starting at a running speed of 8.5 km.h^-1^, which was then increased by 0.5 km.h^-1^ every minute. The running pace was regulated by a prerecorded audiotape, signaling when participants needed to be at one or the other end of the 20-m course. Participants were encouraged to complete as many stages of the MSST as possible, and the test was terminated when they were unable to maintain the prescribed pace. Specifically, participants were given a warning the first time they were behind the sound signal (∼1 m was allowed), and MSST was immediately stopped on the second warning. Total duration of MSST was recorded [7].

#### Test to Exhaustion Specific to Tennis (TEST)

**Fig 1:**
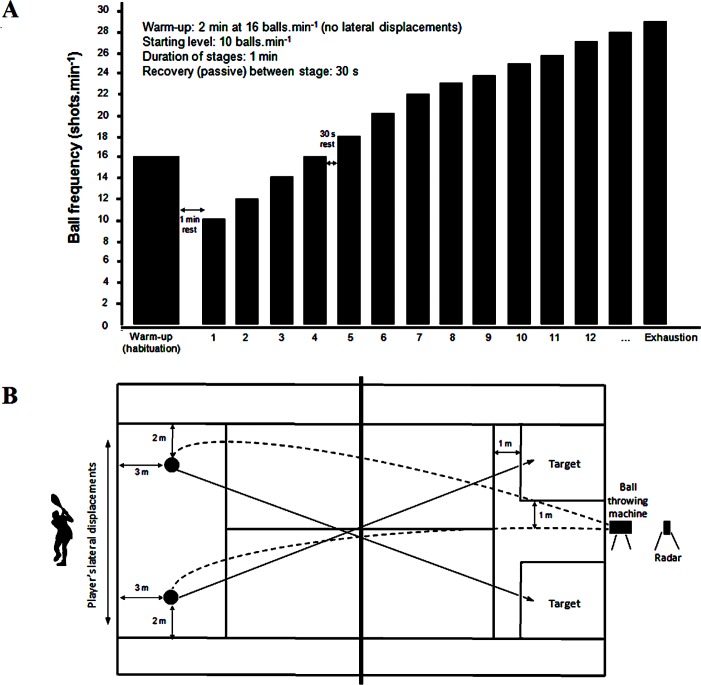
TEST design (A) and schematic setting (B) for the Test to Exhaustion Specific to Tennis (TEST).

TEST procedure consisted of hitting balls thrown at constant velocity (mean: 86 km.h^-1^; coefficient of variation for ball speed = 1.7% and 1.5% for right and left corners of the baseline, respectively, alternating forehand and backhand strokes (Fig 1) by a ‘Hightof’ ball machine. Players had to hit balls cross-court in a prescribed pattern (i.e., topspin drive), while the landing point for thrown balls was set 3 meters in front of baseline (Fig 1). Slice strokes were not allowed because of their potential influence on ball positioning and therefore on TEST performance and associated physiological responses.

A standardized warm-up preceded TEST. It consisted of cycling an ergocycle (75W – 100W), for 10 min. TEST started with a 2-min ‘habituation’ phase where a BF of 16 shots.min^-1^ with balls thrown to the central area of the court (minimal lateral displacement) was adopted. After one minute of passive rest, the main test procedure begun: a BF of 10 shots.min^-1^ was first selected, which was then increased by 2 shots.min^-1^ every minute until the stage corresponding to a BF of 22 shots.min^-1^. From there, increment in BF was set at +1 shots min^1^ (Fig 1). After each 1-min stage, a 30-s passive recovery break (quiet standing) was implemented.

Players were told to “hit the ball with the best possible velocity/accuracy ratio”. Stroke involvement was motivated by ‘live’ (immediate) feedback. Specifically, players were informed about inappropriate ball velocities (ball velocity < 80 km.h^-1^) and precision (30% of balls landing outside the target zone) at the end of each stage completed. Ball speed was measured with a Solstice1 radar (Hightof’^®^, France). To ensure standardized playing conditions, a minimum of 100 balls (Roland Garros^®^) was used every 6 tests.

TEST ended with player’s voluntary exhaustion or was stopped by the researchers if: i) the players felt exhausted or failed to hit the ball twice in a row or ii) the player was no longer able to perform strokes with an acceptable execution technique and a demise in velocity/precision, as determined by experienced coaches, (i.e., national level coaches with > 15 years of experience at the elite level) through subjective observation. Specifically, participants were given a warning the first time they disrespect the rules, while they were stopped on the second warning. Performance was measured as the total test duration.

### Physiological measurements

During both tests the following ventilatory breath-by-breath gas exchange and five-second heart rate (Suunto Ambit2, Vantaa, Finland) values were continuously recorded using the Metamax II CPX system (Cortex, Leipzig, Germany): oxygen uptake ($\dot{\text{V}}$O_2_), carbon dioxide production ($\dot{\text{V}}$CO_2_), respiratory exchange ratio (= $\dot{\text{V}}$CO_2_. $\dot{\text{V}}$O_2_^-1^), and minute ventilation ($\dot{\text{V}}$E). Gas and volume calibration of the measurement device were performed before each test according to manufacturer’ instructions.

The American College of Sports Medicine [18] recommends four criteria to determine maximal effort during incremental tests: i) $\dot{\text{V}}$O_2max_ plateau defined as an increase of less than 1.5 mL.min^-1^.kg^-1^ despite progressive increases in exercise intensity, ii) a final respiratory exchange ratio of 1.1 or above, iii) a final heart rate above 95% of the age related heart rate_max_, and iv) a final blood lactate concentrations above 8 mmol.L^-1^ [18]. Ratings of perceived exertion were recorded using the Borg 6–20 scale at the end of each test. Furthermore, 25 μl capillary blood samples were taken from fingertip and analysed for blood lactate concentrations by using the lactate Pro (LT-1710, Arkray, Japan) [19] portable analyser at the beginning and 15 s after exhaustion.

### Detection of respiratory compensation point (RCP)

RCP detection was done by analyzing the points of change in slope (breaks in linearity) of ventilatory parameters [11,20,21]. RCP was determined using the criteria of an increase in $\dot{\text{V}}$E/ $\dot{\text{V}}$O_2_ with no increase in $\dot{\text{V}}$E/$\dot{\text{V}}$CO_2_ and departure from the linearity of $\dot{\text{V}}$E. All assessments of the RCP were made by visual inspection of graphs of time plotted against each relevant respiratory variable measured during testing. All visual inspections were carried out by two experienced exercise physiologists. The results were then compared and averaged. The difference in the individual determinations of RCP was < 3%.

### Players’ ranking

The international tennis ranking (ATP, WTA and ITF junior) was used to rank players from 1 to 27 in our population sample, with all male players achieving a better ranking than any of the woman players. Players competitive level was confirmed by the professional national coaches of the French Tennis Federation.

### Data analysis

In both tests, the gas samples were averaged every 30 s for further analysis, and the highest values for $\dot{\text{V}}$O_2_ and heart rate over 30 s were regarded as $\dot{\text{V}}$O_2max_ and heart rate_max_. Each physiological variable corresponding to RCP and maximal load was expressed in absolute terms. Physiological variables were then interpolated and further compared using time frames corresponding to 10% of the total duration of each test.

### Statistical analysis

Mean (±SD) was calculated for all variables. Data obtained at RCP and maximal load were compared between MSST and TEST using paired sample-t tests. $\dot{\text{V}}$*O*_*2*_*,* $\dot{\text{V}}$*E,* and heart rate curves were compared using a two-way repeated measures analysis of variance [Condition: (MSST vs. TEST) x Time: (10%, 20% … 100% of test duration)]. However, when the normality test failed, a Mann-Whitney rank sum test was performed between tests at each time interval. The Bonferroni test was used for post hoc comparisons. Finally, performance and $\dot{\text{V}}$O_2_ corresponding to RCP and maximal load were also correlated with the competitive level (ranking) of the players using Pearson rank order correlation for both tests. Data were tested for normality (Shapiro–Wilk test) and equality of variance (Fisher–Snedecor F-test). Where significant effects were established, pairwise differences were identified using the Bonferroni post hoc analysis procedure adjusted for multiple comparisons.

The following criteria were adopted to interpret the magnitude of r: <0.1, trivial; 0.1–0.3, small; 0.3–0.5, moderate; 0.5–0.7, large; 0.7–0.9, very large; and 0.9–1.0, almost perfect [22]. Statistical significance was accepted at p ≤ 0.05. The statistical analyses were performed using SigmaStat 3.5 software.

## Results

### Test performance

Test duration was longer (p = 0.001) for TEST (908 ± 94 s) compared with MSST (665 ± 100 s). The number of players who satisfied the criteria for maximum effort for each test is displayed in Table 1.

**Table 1:** Number of athletes who satisfied the criteria for a maximum effort.

	TEST	MSST
VO_2_	27 (100%)	21 (78%)
RER	27 (100%)	25 (96%)
HR	21 (78%)	23 (85%)
La^-^	22 (81%)	24 (89%)

RER, respiratory exchange ratio; HR, heart rate.

### Physiological responses

**Fig 2:**
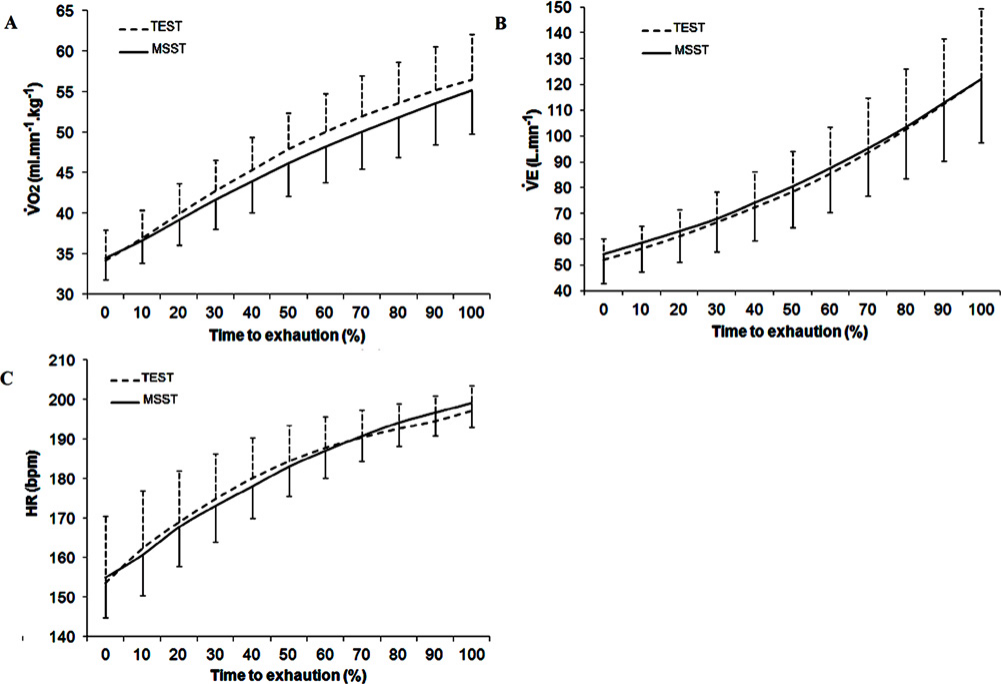
**Oxygen uptake ($\dot{\text{V}}$O_2_, A), minute ventilation ($\dot{\text{V}}$E, B), and heart rate (HR, C) during TEST and MSST in tennis players (n = 27).** Data are expressed relatively (10% frames) as a function of total test duration.

At submaximal load (RCP), higher $\dot{\text{V}}$O_2_ values were recorded for TEST compared to MSST (+5.2%; p = 0.05) (Table 2B). However, $\dot{\text{V}}$E and heart rate values did not differ between tests. When data were compared at similar relative exercise durations (10% increments), there were no differences between tests (i.e., all p values > 0.05 for condition x time interaction effects) for any physiological variable (Fig 2). At maximal loads, $\dot{\text{V}}$O_2max_, $\dot{\text{V}}$E_max_, heart rate_max_, blood lactate concentration and ratings of perceived exertion values did not differ between TEST and MSST (Table 2A).

**Table 2:** A: maximal levels and significant differences in maximal oxygen uptake (VO_2max_), ventilation (VE_max_), heart rate (HR_max_), lactates ([La^-^_max_]) and ratings of perceived exertion scale (RPE) between the specific tennis test (TEST) and the 20m shuttle run test (MSST); B: levels of VO_2_, VE and HR at respiratory compensation point (RCP) between TEST and MSST. Data are expressed as mean ± standard deviation. P ≤ 0.05 for differences between TEST and MSST.

A: Maximal level	TEST	MSST	p
VO_2max_ (mL.mn^-1^.kg^-1^)	56.5 ± 5.6	55.2 ± 5.5	0.25
VE_max_ (L.mn^-1^)	121.1 ± 26.7	122.1 ± 24.9	0.77
HR_max_ (bpm)	197.2 ± 6.5	199.2 ± 6.3	0.24
[La^-^_max_] (mmoles.L^-1^)	9.7 ± 3.3	9.6 ± 2.3	0.92
RPE	17.1 ± 1.4	16.4 ± 1.6	0.18
**B: RCP level**			
VO_2_ (mL.mn^-1^.kg^-1^)	50.1 ± 4.7	47.5 ± 4.3	0.05
VE (L.mn^-1^)	85.4 ± 18.2	84.2 ± 16.5	0.97
HR (bpm)	187.7 ± 7.9	185.2 ± 7.3	0.66

### Correlations of test performance and physiological responses with competitive level

**Fig 3:**
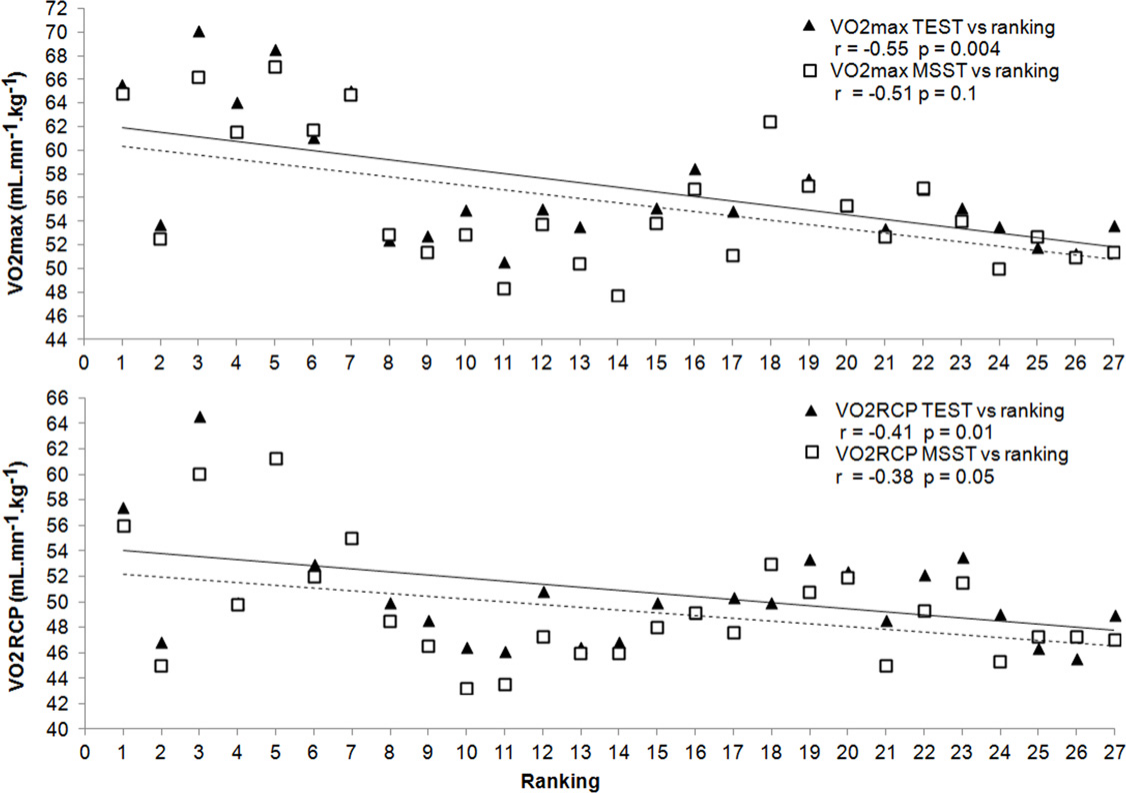
**Relation of players’ ranking (1 to 27) with maximal oxygen uptake ($\dot{\text{V}}$O_2max_, A), and oxygen uptake at the respiratory compensation point ($\dot{\text{V}}$O_2RCP_, B).** Dotted and plain lines represent TEST and MSST, respectively.

The players’ ranking was negatively correlated with $\dot{\text{V}}$O_2max_ for both TEST and MSST (r = -0.55; p = 0.04 and -0.51; p = 0.1) (Fig 3A). Significant Pearson’s r coefficients were observed between ranking and $\dot{\text{V}}$O_2_ obtained at RCP and expressed both with absolute (TEST: r = -0.41, p = 0.01; MSST: r = -0.38, p = 0.05) values (Fig 3B). Finally, MSST (r = -0.46, p = 0.01), but not TEST (r = 0.04, p = 0.8), duration correlated negatively with competitive level.

## Discussion

### TEST design and methodological considerations

In order to better standardize parameters influencing $\dot{\text{V}}$O_2_ response, we decided not to stimulate the great variety of playing situations during a tennis match (i.e., low level of uncertainty in the protocol so that progress cannot be associated with a learning process) and maintain constant ball quality produced by the machine. Indeed, Bekraoui et al. [14] observed that offensive strokes mobilize 6.5% more energy than defense ones. Furthermore, energy required for hitting backhands is 7% higher than for forehands in club-level players. Fernandez-Fernandez et al. [13] also found higher energy cost for forehands (18.5 kcal.min^-1^) compared to backhands (16.8 kcal.min^-1^) for flat, but not topspin, strikes. While the number of forehands and backhands was kept similar during TEST, the selected ball speed (mean: 86 km.h^-1^) corresponds well to what has already been recorded during tennis drills where players were instructed to hit balls with lift [13]. While the consistency in speed and length of balls thrown by a tennis coach is questionable even for experienced coaches [23], the use of an accurate and reliable throwing machine was a strong methodological point to standardize our experimental conditions. Unlike Fargeas-Gluck and Leger [23], we believe that the determination of the running speed obtained from TEST is not a discriminating criterion in that energy expenditure is related to the level of engagement in strikes. During MSST, directional changes (180°) after 20-m shuttle runs are likely more intense than during TEST after shorter displacements ranging between 4 and 8 m.

Because it is unlikely that players can maintain elevated strokes efficiency during several incremental steps using long exercise stages (> 1 min), especially in the absence of intervening recovery periods, TEST resembles more a tennis-specific procedure. During TEST, the duration of stages was set to 1 min interspersed by 30-s recovery periods. Because it is commonly thought that it would allow a more reliable determination of ventilatory and lactate thresholds [5], stage durations of 2–3 min and a continuous mode of exercise have often been used in available test procedures [9,12], Nonetheless, Balmer et al. [24] showed that the maximum power determined by one minute increments in load are also highly reliable [24]. Such stage durations have been adopted in other incremental tennis-specific procedures (NAVTEN, [23]) and Hit & Turn Tennis Test [10], while even shorter stage durations have been used elsewhere [11]. Moreover, relatively short protocols (< 20 min), as used here, allow reliable determination of $\dot{\text{V}}$O_2max_ with 1-min stages [25]. Some previous studies have shown that hitting balls during specific aerobic training may have deleterious effects on forehand and backhand technique in teenage developing players [26,27]. At elite level, the technical skills are paramount and have to be trained on a systematic basis, which also include aerobic type sessions. For this purpose, TEST is of high practical interest.

### Submaximal loads

When designing an incremental test procedure, it is important that the first few stages are not too demanding physiologically in order not to overtax the anaerobic process [28]. Observed correlations for physiological parameters between the two tests and the progressive increases in physiological load are therefore important. At RCP, we found higher$\dot{\text{V}}$O_2_ values for TEST compared to MSST. It underlines that during a specific activity, players might tolerate higher metabolic stress before activating the RCP. This may relate to the fact that players probably felt more comfortable performing on-court movements (i.e., displacements with specific footwork and ball hitting) than during a shuttle running, including sharper (and potentially more demanding) directional changes and limited upper body involvement. In support, performance, physiological and perceptual responses during repeated sprints with changes of direction are angle-dependent [29]. Interestingly, players with higher aerobic fitness compete at relatively lower exercise intensities [30]. Indeed, Baiget et al. [30] observed that players during competition scenarios spent more than 75% of the time in their low-intensity zone (under first ventilatory threshold), with less than 25% of the time spent at moderate-to-high intensities (between first and second ventilatory threshold). Evaluating specific aerobic fitness including elements of the game is therefore useful as it may determine the metabolic intensity that players can sustain throughout a game.

### Maximal loads

It is known that incremental tests lasting between 5 and 26 minutes are relevant for eliciting valid $\dot{\text{V}}$O_2 max_ values [31] and that relatively short protocols (< 20 min) with 1-min stages, as used here, also allow reliable $\dot{\text{V}}$O_2max_ determination [25]. During TEST, all participants fulfilled the criteria of plateau in $\dot{\text{V}}$O_2_ with the majority of players also satisfying other criteria for maximal efforts, indicating that they were effectively exhausted by this new test procedure. Measuring $\dot{\text{V}}$O_2max_ using TEST is relevant and may be used to prescribe aerobic exercise in a context more appropriate to game play than MSST. In line with previous tennis field-tests literature (The Hit & Turn Tennis Test) [10], VO2max values reached on both tests were higher in males compared to females. Comparable values of other physiological variables and blood lactate concentration reached at exhaustion between the two tests further reinforce the usefulness of TEST in elite players.

Our findings, however, differ from Girard et al [11] and Smekal et al. [12]. On the one hand, Girard et al. [11] postulated that $\dot{\text{V}}$O_2max_ values derived from laboratory testing (treadmill) may not be relevant for accurately estimating aerobic fitness in tennis players since $\dot{\text{V}}$O_2max_ values were significantly lower on a treadmill *vs*. field test (58.9 ± 5.3 vs. 63.8 ± 5.7 mL^-1^.min.kg^-1^). On the other hand, Smekal et al. [12] found higher $\dot{\text{V}}$O_2max_ values with treadmill (58.3 ± 4.3 *vs*. 52.4 ± 3.7 mL^-1^.min.kg^-1^) compared to on-court incremental tests. Discrepancies between our results and above findings may well relate to the details of the various tests and to the characteristics of tested players (i.e., level, age, gender). Furthermore, while TEST was compared to MSST in our study, previously developed test procedures have been compared with treadmill protocols that are in nature not specific to tennis (no directional changes or specific upper limbs involvement).

If possessing a high $\dot{\text{V}}$O_2max_ is certainly not a discriminating factor for tennis performance as opposed to continuous aerobic activities (i.e., running, cycling), Bergeron et al. [32] indicated that players with a well-developed $\dot{\text{V}}$O_2max_ would better sustain cardiovascular load and improve their recovery between points. In support, a strong inverse relationship between $\dot{\text{V}}$O_2max_ and ATP entry ranking over time in a professional tennis player has been reported [33]. That said, Kovacs [34] postulated that for a top player, it is important to have a $\dot{\text{V}}$O_2max_ > 50 mL.min^-1^.kg^-1^ for women et 55 mL.min^-1^.kg^-1^ for men but that higher$\dot{\text{V}}$O_2max_ (> 65 mL.min^-1^.kg^-1^) does not further improve on-court performance against a $\dot{\text{V}}$O_2max_ of ∼55–60 mL.min^-1^.kg^-1^. This is reflected here where the best French junior players displayed averaged $\dot{\text{V}}$O_2max_ values of 56.5 ± 5.6 mL.min^-1^.kg^-1^. This is because performance in tennis is largely dependent on the technical, tactical and motor control/coordination aspects.

### Relationships of physiological response and test performance with competitive ranking

There was no correlation between the ranking of players and TEST duration (r = - 0.06), unlike in the study by Girard et al. [15] who did report a strong correlation (r = - 0.96) for elite players tested on a squash specific graded test. Compared to Girard et al. [15], our population sample was larger (n = 7 *vs*. 27 participants) with also a more homogeneous sample of players, so that any significant relationship would be more difficult to distinguish. Furthermore, while a significant relationship between MSST duration and competitive ranking (r = -0.46, p < 0.05) has been observed, the lack of significant relationship for TEST may be because of the involvement with stroke hitting. This highly individual characteristic may lead to variable energy cost depending on players’ game style and technique.

In our study, the moderate correlations of competitive ranking with $\dot{\text{V}}$O_2max_ (r = -0.55) and $\dot{\text{V}}$O_2_ at RCP (r = -0.47), while rather similar relationships also occurred for MSST(r = -0.41 and r = -0.38), reinforce the interest of implementing TEST. In partial agreement with these findings, Baiget et al. [9] indicated that large part of the variability in the 38 competitive players that they have tested on a relatively similar incremental test could be explained by time to exhaustion and physiological parameters (RCP and maximal load). However, in the present study and in the one by Baiget et al. [9], correlations were only moderate, which reflect the complexity of tennis performance depending on a mixture of factors. It would be useful in future studies to relate physiological variables derived from TEST with indices of match intensity or fatigue resistance.

### Practical implications

Coaches may sometimes be reluctant using new technologies, as they believe it may negatively influence performance of their players (extra psycho-physiological stress). In this instance, observation of consistent performance and associated physiological variables when carrying or not a portable gas analyzer during on-court testing is encouraging [9]; wearing a mask may even induce an extra motivation (i.e Hawthorne effect) [35]. That said, TEST can also be practised without wearing a gas analyser to evaluate the ability of players to work in a fatigued state from the knowledge of target intensities (i.e., a given stage or the BF corresponding to players’ RCP) to focus on technique stabilization. Hence, recent publications [13,36–39] highlight the importance of an integrated physical work on the tennis court, by monitoring physiological responses during the completion of specific on-court drills.

TEST can also be used to ascertain the effects of ergogenic aids or of a training intervention. Interestingly, Fernandez-Fernandez et al. [37] found a significant increase in $\dot{\text{V}}$O_2max_ after tennis players both followed high-intensity interval training and repeated sprint training modalities, yet a treadmill test was implemented. In order to improve test specificity, TEST could be used, for instance to assess the effects of innovative training methods such as repeated sprinting in hypoxia [40] likely to maximize aerobic fitness in tennis players compared to similar training at sea level.

Adopting an intermittent exercise pattern is an important methodological difference with existing tests [9,12]. First, 30-s rest periods between stages are relatively close to between-points recoveries during official competitions (∼20 s), potentially allowing a window to measure blood lactate concentration. Second, the technical quality of the stroke is paramount to reach the highest level and a test allowing actual ball hitting is probably of higher interest for coaches. Hence, standardized evaluations of ball velocity/accuracy under various fatigue conditions are made possible.

## Conclusion

Building on recent efforts made to develop field tests in tennis, we introduce here a Test to Exhaustion Specific to Tennis (the so-called TEST) including elements of game play (i.e., specific footwork, ball hitting, intermittent activity) and compared performance and physiological responses to a widely used field procedure (MSST). At submaximal intensity (RCP) $\dot{\text{V}}$O_2,_ but not $\dot{\text{V}}$E and heart rate, values were higher for TEST compared to MSST. However, load increment and physiological responses at maximal load were similar between the two tests. Results also indicate that $\dot{\text{V}}$O_2_ values both at submaximal and maximal loads reached during TEST and MSST are moderate predictors of players competitive ranking. Test–retest are still needed to guarantee the excellent reproducibility of performance and physiological responses. In doing so, testing players of various standards or age groups using TEST would allow to relate players to their peers (chronological and biological age) through the determination of age-group percentiles, for instance as done for the Hit & Run Tennis Test (Ferrauti et al., 2011). In order to identify players whose $\dot{\text{V}}$O2max is sufficiently high in their age group and meeting the physiological demands of senior match play, building normative tables for different groups may also be informative.
